# Functional Analysis of CPSF30 in *Nilaparvata lugens* Using RNA Interference Reveals Its Essential Role in Development and Survival

**DOI:** 10.3390/insects15110860

**Published:** 2024-11-03

**Authors:** Shengli Jing, Jing Yang, Yali Liu, Feifei Wang, Fang Zheng, Aobo Ren, Bingbing Yu, Yue Zhao, Bing Jia, Ruixian Chen, Bin Yu, Qingsong Liu, Jingang Xu

**Affiliations:** 1 College of Life Sciences, Xinyang Normal University, Xinyang 464000, China; shljing@xynu.edu.cn (S.J.); jingyang202409@163.com (J.Y.); liuyali_020601@163.com (Y.L.); ffwang202203@163.com (F.W.); zf15837634522@163.com (F.Z.); renaobo202202@163.com (A.R.); yubingbing2021@163.com (B.Y.); zhaoyue040721@126.com (Y.Z.); jia825285@163.com (B.J.); chenruixian2002@163.com (R.C.); yubin_2015@126.com (B.Y.); qingsongliu@henu.edu.cn (Q.L.); 2 State Key Laboratory of Cotton Bio-Breeding and Integrated Utilization, State Key Laboratory of Crop Stress Adaptation and Improvement, Key Laboratory of Plant Stress Biology, School of Life Sciences, Henan University, Kaifeng 475004, China

**Keywords:** *Nilaparvata lugens*, *NlCPSF30*, gene function, lethal phenotypes, RNA interference

## Abstract

The brown planthopper (*Nilaparvata lugens*) is a major pest threatening global rice production, significantly reducing yields annually. As *N. lugens* increasingly develops resistance to conventional control methods, such as chemical pesticides, there is an urgent need for innovative and sustainable pest management strategies. Cleavage and Polyadenylation Specificity Factor 30 (CPSF30) is a key protein involved in mRNA 3′ end processing, yet its function in *N. lugens* remains poorly understood. This study aims to elucidate the role of CPSF30 in the growth and development of *N. lugens* and evaluate its potential as a target for RNA interference (RNAi)-based pest control strategies. We cloned and characterized the cDNA sequence of *NlCPSF30*, which encodes a protein of 341 amino acids containing five CCCH zinc-finger domains and two CCHC zinc-knuckle domains. Sequence alignment revealed that NlCPSF30 is highly conserved among insect species, particularly in the zinc-finger domains essential for RNA binding and processing. Phylogenetic analysis showed that NlCPSF30 is closely related to CPSF30 proteins from other hemipteran species. Expression analysis indicated that *NlCPSF30* is most highly expressed in the fat body and during the adult stage, with significantly higher expression in females than in males. RNAi-mediated silencing of *NlCPSF30* in third-instar nymphs resulted in severe phenotypic abnormalities, including disrupted molting and increased mortality following injection of double-stranded RNA (dsRNA) targeting *NlCPSF30*. Moreover, it influenced the expression of genes associated with hormone regulation, namely *NlHry*, *NlE93*, and *NlKr-h1*. These results suggest that *NlCPSF30* is integral to critical physiological processes, with its disruption leading to increased mortality. Our findings identify *NlCPSF30* as an essential gene for *N. lugens’* survival and a promising target for RNAi-based pest management strategies. This study provides a valuable molecular target and theoretical insights for developing RNAi-based control methods against *N. lugens*.

## Simple Summary

The brown planthopper (*Nilaparvata lugens*) is a major pest threatening rice crops, especially in Asia and Africa, causing damage and transmitting harmful viruses. Current control strategies, such as the use of chemical pesticides, are becoming increasingly ineffective due to the development of resistance in pest populations. RNA interference (RNAi) is a promising alternative for silencing essential genes in pests. This study focuses on the *NlCPSF30* gene (homolog of mammalian CPSF30), which is crucial for mRNA processing in brown planthoppers. By using RNAi to knock down the *NlCPSF30* gene, we observed marked reductions in survival rates and notable developmental defects in the pests. These findings highlight the potential of targeting *NlCPSF30* for developing RNAi-based pest control strategies.

## 1. Introduction

Rice (*Oryza sativa*) is a vital staple crop, particularly in Asia and Africa, but its production is severely threatened by pests such as the brown planthopper (*Nilaparvata lugens*, BPH), a member of the Hemiptera order. This insect is a major pest that causes direct damage to rice plants and transmits viruses like rice stripe virus and rice grassy stunt virus [1,2,3]. Conventional control methods, including chemical pesticides and resistant crop varieties, are becoming less effective due to the pest’s rapid adaptation and the development of pesticide resistance and virulence of this pest [4]. Therefore, developing innovative and sustainable pest management strategies is imperative.

RNA interference (RNAi) has emerged as a promising approach for pest control due to its ability to specifically silence target genes with high precision [5]. This technique harnesses the power of double-stranded RNA (dsRNA), which is introduced into the cells of target organisms. This leads to the effective suppression of gene function, either by completely silencing the gene or significantly reducing its activity. It is worth noting that RNAi serves as a natural regulatory mechanism against viral infections and gene expression in numerous eukaryotic organisms [6]. Therefore, artificial synthesis of dsRNA of specific genes of pests can be used to introduce them into their cells and hinder their growth even though it can result in death. Based on the origin of dsRNA synthesis, two main strategies for pest control are sprayable dsRNA biopesticides and plant-mediated RNAi strategies. The first sprayable dsRNA biopesticide product, Ledprona [7], was approved by the United States Environmental Protection Agency (EPA) in 2023 (U.S. EPA, 2023). Similarly, the first plant-mediated RNAi product, MON87411, was approved by the U.S. EPA in 2017 (U.S. EPA, 2017) [8]. Ledprona is a long dsRNA that targets the *proteasome subunit beta 5* (ds*PSMB5*) of the Colorado potato beetle [7]. MON87411, on the other hand, is a transgenic maize variety that expresses a dsRNA targeting *sucrose non-fermenting 7* (*DvSnf7*) of the western corn rootworm [8]. These commercially available products represent a novel class of RNAi-based biopesticides for pest control. As a result, numerous studies have focused on investigating the gene functions of various agricultural pests using RNAi approaches, particularly Hemiptera pests. For instance, *Sogatella furcifera* exhibited *chitin deacetylase* [9] and *vitellogenin-like1* [10] genes, *N. lugens* displayed *peroxisomal factor NlPEX14* [11], *groucho* and *groucho1-like* [12] genes, *Megoura viciae* possessed the *tyrosine hydroxylase* [13] gene, and *Diaphorina citri* contained the *hexokinase* [14] gene. Silencing these genes led to abnormal development, increased mortality, and impaired reproduction, thus indicating their potential as target genes for controlling Hemiptera pests. Consequently, it is crucial to screen and identify target genes with significant functions and high RNAi efficiency to enhance pest control measures.

In eukaryotes, the proper processing of precursor mRNA (pre-mRNA) is critical for generating mature mRNA, involving multiple protein complexes responsible for 5′ capping, 3′ polyadenylation, and RNA splicing [15,16]. The 3′ end processing, involving cleavage and polyadenylation, is essential for mRNA stability and function [17]. Key proteins, including cleavage and polyadenylation specificity factor (CPSF), cleavage stimulation factor (CstF), cleavage factors (CFI and CFII), and poly(A) polymerase (PAP), coordinate these processes. These factors recognize the polyadenylation signal, cleave the pre-mRNA, and add a poly-A tail, enhancing mRNA stability and translation efficiency [18,19].

The Cleavage and Polyadenylation Specificity Factor 30 (CPSF30) is essential for 3′ end mRNA processing, as it recognizes specific sequences within mRNA to facilitate pre-mRNA cleavage and polyadenylation, thereby influencing mRNA stability and translational efficiency [20,21]. In various organisms, including humans, *Arabidopsis*, and yeast, CPSF30 possesses three to five CCCH zinc-finger (ZF) domains at the N-terminal. Among these domains, two ZF domains specifically recognize the AAUAAA poly(A) signal and bind to RNA. The C-terminal of CPSF30 may or may not contain a CCHC zinc-knuckle domain, which is responsible for recognizing and binding to polyuracil RNA [22,23,24,25]. In model organisms like yeast [26], *Drosophila* [17,27], and *Arabidopsis thaliana* [28], CPSF30 has been established as a critical regulator of gene expression. Loss of CPSF30 function in these organisms often leads to abnormal development and even death. However, the role of CPSF30 in agricultural pests, such as the brown planthopper, remains largely unexplored.

Given the fundamental role of CPSF30 in these organisms, this study hypothesizes that NlCPSF30 (homolog of mammalian CPSF30) is also vital for the brown planthopper and investigates its potential as an RNAi target. We cloned the coding region of the *NlCPSF30* gene from the brown planthopper, conducted bioinformatics analyses, and examined the spatiotemporal expression patterns of *NlCPSF30* using quantitative real-time PCR. Subsequently, we employed RNAi to silence the *NlCPSF30* gene in the brown planthopper and investigated its role in the growth and development of the pest. Our findings provide new insights into the molecular mechanisms of brown planthopper biology and identify *NlCPSF30* as a potential target for RNAi-based pest control strategies.

## 2. Materials and Methods

### 2.1. Insects

The *N. lugens* used in this study was originally sourced from Wuhan University, Hubei, China. The insects were reared on TN1 (Taichung Native 1), a rice variety susceptible to brown planthopper. The rearing conditions were as follows: a temperature of 26 ± 2 °C, a photoperiod of 16 h of light and 8 h of darkness, and a relative humidity of approximately 65 ± 5%.

### 2.2. Total RNA Extraction and cDNA Synthesis

To clone the *NlCPSF30* gene sequence, total RNA was extracted from thirty fourth-instar nymphs of *N. lugens* using the RNAiso Plus Kit (Takara, Dalian, China), according to the manufacturer’s protocol. RNA integrity was assessed by 1% agarose gel electrophoresis, and the RNA concentration and purity were measured using a Nanodrop 2000 spectrophotometer (Thermo Fisher Scientific, Waltham, MA, USA). cDNA was synthesized via reverse transcription using the PrimeScript™ RT Reagent Kit with gDNA Eraser (Takara, Dalian, China), following the manufacturer’s instructions [29].

### 2.3. Cloning and Sequencing of the NlCPSF30 Gene

The *NlCPSF30* gene (a homolog of mammalian CPSF30) sequence (XM_022344588) was retrieved from the *N. lugens* genome database on the National Center for Biotechnology Information (NCBI) website (http://www.ncbi.nlm.nih.gov/) (accessed on 15 August 2024). Gene-specific primers were designed using Primer Premier 5.0 software based on the downloaded sequence (Table 1). The cDNA was amplified via polymerase chain reaction (PCR) using La Taq (Takara, Dalian, China) and gene-specific primers. The PCR conditions consisted of an initial denaturation step at 95 °C for 5 min, followed by 35 cycles of denaturation at 95 °C for 30 s, annealing at 55 °C for 30 s, and extension at 72 °C for 1 min. The amplification process concluded with a final extension step at 72 °C for 10 min. PCR products were ligated into the pMD-18T vector (Takara, Dalian, China) for cloning. The recombinant plasmid was transformed into competent cells, and positive clones were selected for sequencing by Wuhan HeTaiQing Biological Company. The complete coding sequence of the *NlCPSF30* gene was then obtained.

### 2.4. Sequence and Phylogenetic Analysis

Homologous CPSF30 proteins from other insect species were retrieved from the NCBI database (https://www.ncbi.nlm.nih.gov/protein) (accessed on 20 August 2024). The structural domains of these homologous proteins were predicted using the SMART online tool (http://smart.embl-heidelberg.de/) (accessed on 20 August 2024). A phylogenetic tree was constructed using the neighbor-joining (NJ) method in MEGA5.0 software [30], based on the NlCPSF30 protein sequence and homologous CPSF30 sequences from 42 plant, mammal, and insect species. Bootstrap analysis was performed with 1000 replicates, and the results were expressed as percentages. The accession numbers of the sequences used for the multiple sequence alignment and phylogenetic analysis are listed in Table S1.

### 2.5. Expression Pattern Analysis of NlCPSF30

The spatial and temporal expression of *NlCPSF30* was assessed using quantitative real-time PCR (qRT-PCR). For temporal expression analysis, samples were collected at different developmental stages: eggs (*n* = 240), first-instar (*n* = 180), second-instar (*n* = 120), third-instar (*n* = 60), fourth-instar (*n* = 30), fifth-instar (*n* = 15), and female (*n* = 5) and male adults (*n* = 10) at 3 days post-emergence. For spatial expression analysis, at least 20 female and 20 male adults (1–3 days post-emergence) were collected. Antennae, salivary glands, gut, leg, ovaries, and fat body were dissected from the female adults, while testes were dissected from the male adults. All samples were collected in triplicate for biological replication. Total RNAs were extracted from all developmental stages and tissues and reverse-transcribed into cDNA. Specific primers for qRT-PCR were designed based on the cloned gene sequence of *NlCPSF30* using Primer Premier 5.0 software (Table 1). The *18S ribosomal RNA* gene (*18S rRNA*, JN662398) served as the internal reference. qRT-PCR was performed using the CFX96™ Real-Time PCR Detection System (Bio-Rad, Philadelphia, PA, USA), and each assay included three technical replicates. Expression differences of *NlCPSF30* across the developmental stages and tissues were analyzed using one-way ANOVA and quantified using the 2^−ΔΔCt^ (Ct: cycle threshold) method [31].

### 2.6. Double-Stranded RNA (dsRNA) Synthesis

The dsRNAs were synthesized to achieve the knockdown of *NlCPSF30*. To ensure reliable detection of the target gene at the transcriptional level, the fragment used for dsRNA synthesis was designed to avoid overlapping with the qRT-PCR detection region. The dsRNA-specific primers containing T7 promoter sequences were designed based on the cloned sequence of the *NlCPSF30* gene, with the *green fluorescent protein* (*GFP*, GenBank accession number: MN114103) gene serving as a control (Table 1). The cloned segments of *NlCPSF30* and *GFP* were inserted into plasmids, which were purified using the Plasmid Mini-Extraction Kit (Biotek, Guangzhou, China) according to the manufacturer’s instructions. The purified products were then used to synthesize ds*NlCPSF30* and ds*GFP* using the MEG Ascript T7 High Yield Transcription Kit (Ambion, Austin, TX, USA) following the manufacturer’s guidelines. The quality and concentration of the synthesized dsRNAs were assessed via 1% agarose gel electrophoresis and a Nanodrop 2000 spectrophotometer (Thermo Fisher Scientific, USA). The final dsRNA concentration was adjusted to 1000 ng/μL using DEPC-treated water.

### 2.7. RNAi

Approximately 140 third-instar nymphs of the brown planthopper were selected for microinjection in each experimental replicate. Specifically, 80 nymphs were injected with ds*NlCPSF30* and 60 with ds*GFP.* The injections were performed using a Nanoliter 2020 injector (World Precision Instrument, Sarasota, FL, USA) following carbon dioxide (CO_2_) anesthesia for 10–15 s. Each nymph was injected with 23 nL of dsRNA (1000 ng/μL) at the mid-line of the thorax [32]. Two hours after injection, the planthoppers were transferred to fresh TN1 rice seedlings for feeding.

To confirm the silencing efficiency of RNAi, five planthoppers were randomly collected from each group at 24 h and 72 h post-injection. The experiment was conducted in four biological replicates to ensure robust data collection. Total RNA was extracted and reverse-transcribed into cDNA. qRT-PCR was conducted to determine the silencing efficiency of *NlCPSF30* following dsRNA injection. Differences in gene silencing efficiency were analyzed using the Student’s *t*-test.

### 2.8. Survival Phenotype Analysis of BPH After RNAi

In each experimental replicate, approximately 140 third-instar nymphs were injected with ds*NlCPSF30* and ds*GFP*, following the procedure outlined above. Survival rates and death phenotypes were observed every 24 h for 10 days. Nearly all planthoppers injected with ds*NlCPSF30* died within this period. Each group included three biological replicates. The proportion of survival planthoppers was calculated, and the data were processed and analyzed accordingly. The mortality phenotypes were photographed using a Leica S8APO stereomicroscope (Leica Microsystems, Wetzlar, Germany).

### 2.9. Effects of NlCPSF30 Knockdown on the Expression of Hormone-Related Genes

To assess the impact of *NlCPSF30* knockdown on hormone-related gene expression, third-instar nymphs were injected with ds*NlCPSF30* and ds*GFP* as the control. After six days post-injection, RNA was extracted from the insects and converted into cDNA through reverse transcription. The expression levels of hormone-related genes (*NlKr-h1*, *NlHry*, and *NlE93*) were conducted using qRT-PCR. To ensure statistical robustness, three biological replicates were performed for each gene.

### 2.10. Data Analysis

Statistical analyses were conducted using SPSS 22.0 software (IBM, New York, NY, USA). Data were presented as means ± SEM. The statistical significance of gene silencing efficiency and the survival rate was assessed using the Student’s *t*-test (* *p* < 0.05; ** *p* < 0.01; *** *p* < 0.001). A one-way ANOVA was followed by Fisher’s least significant difference (LSD) multiple comparison test, and a *p*-value of less than 0.05 was considered statistically significant. The images were captured with a stereomicroscope (Leica S8APO, Wetzlar, Germany).

## 3. Results

### 3.1. Sequence Analysis of NlCPSF30

**Figure 1:**
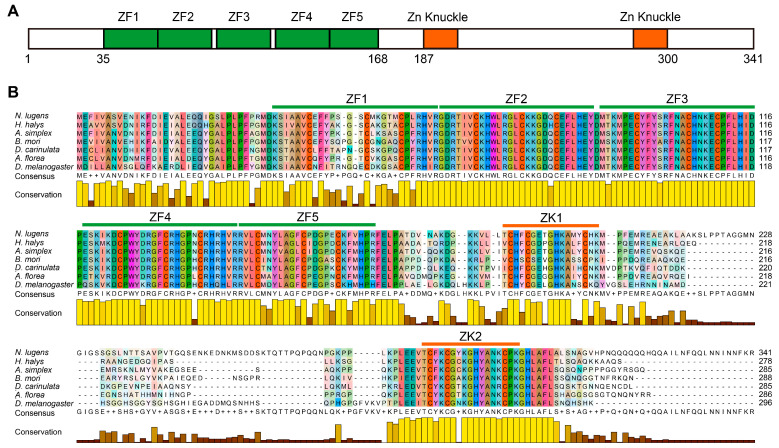
The structures and alignments of amino acid sequences of NlCPSF30 protein. (**A**) Schematic representation of the domain organization in the NlCPSF30 protein. (**B**) Amino acid sequences alignment across six insect orders: *Halyomorpha halys* (Hemiptera), *Anabrus simplex* (Orthoptera), *Bombyx mori* (Lepidoptera), *Diorhabda carinulata* (Coleoptera), *Apis florea* (Hymenoptera), and *Drosophila melanogaster* (Diptera). The green and orange lines represent five CCCH zinc-finger (ZF) domains and two CCHC zinc-knuckle (ZK) domains, respectively.

Using cDNA from BPH nymphs as a template, we amplified a segment of the *NlCPSF30* gene via PCR and sequenced the resulting positive clones. The cloned sequence contained a coding sequence (CDS) of 1026 base pairs (bp) spanning six exons located on chromosome 3. This CDS is predicted to encode a 341-amino acid protein. Sequence analysis revealed that NlCPSF30 contains five zinc-finger domains (amino acids 35–168) at the N-terminus and two zinc-knuckle domains (amino acids 87–203 and 284–300) at the C-terminus (Figure 1). Alignment of the NlCPSF30 protein with homologous CPSF30 proteins from six insect orders demonstrated a high degree of sequence conservation (Figure 1B). While most domains were highly conserved, zinc finger 1 (ZF1) and zinc knuckle 1 (ZK1) exhibited lower conservation than their respective counterparts. Among the insects included in our study, NlCPSF30 showed the highest amino acid identity (62.07%) with the hemipteran insect *Halyomorpha halys*. Conversely, the lowest amino acid identity of 51.45% was observed with the dipteran insect *Drosophila melanogaster* (Table S2). These findings suggest that NlCPSF30 is highly conserved across various insect species.

### 3.2. Phylogenetic Analysis of NlCPSF30

**Figure 2:**
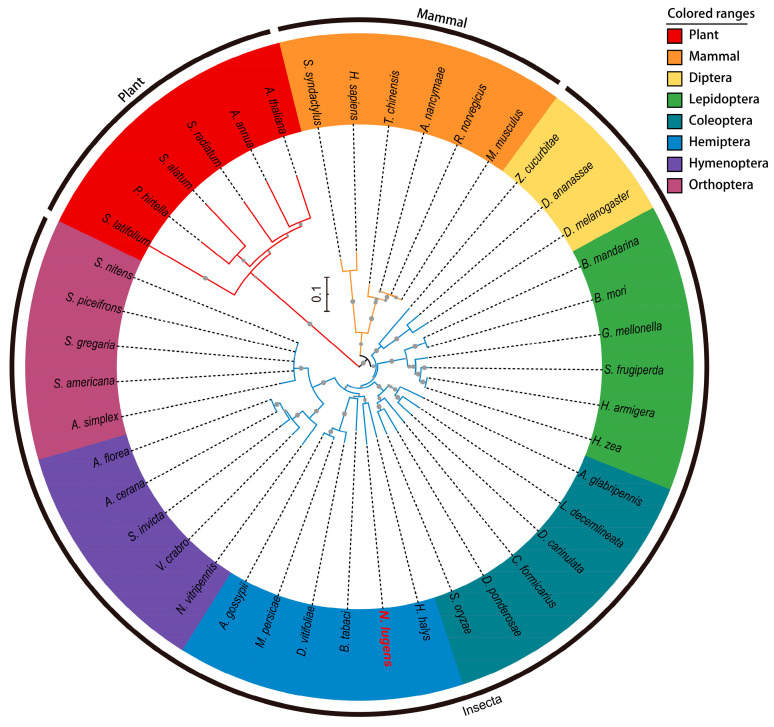
A phylogenetic tree was constructed with NlCPSF30 and 42 orthologs. Different color backgrounds represent the species from specific orders of insects, plants, and mammals. The NlCPSF30 protein of *N. lugens* is indicated in red font.

To elucidate the evolutionary relationship of the NlCPSF30 protein, we conducted an analysis of its amino acid sequence in comparison with orthologs from other species. The sequence was compared with the CPSF30 proteins from 42 species spanning three groups: mammals, insects, and plants (Figure 2). The proteins from the same orders or classes predominantly clustered together in the phylogenetic analysis. Notably, the CPSF30 protein of *N. lugens* exhibited a closer evolutionary relationship with those of other insects than with those of mammals and plants, specifically clustering within the Hemiptera order of insects. The analysis indicated that *N. lugens* is most closely related to *Halyomorpha halys*, another member of the Hemiptera order, with both species clustering within the same clade (Figure 2 and Figure S1).

### 3.3. Spatial and Temporal Expression Analyses of NlCPSF30

**Figure 3:**
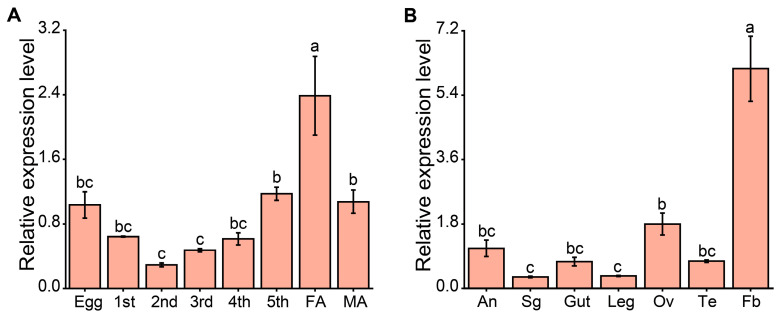
Temporal and spatial expression analyses of *NlCPSF30*. (**A**) Expression analysis of *NlCPSF30* across the developmental stages of the brown planthopper, including eggs, nymphs (first–fifth instars), adult females (FA), and adult males (MA). (**B**) Tissue-specific expression analysis of *NlCPSF30* in different tissues of the brown planthopper: antenna (An), salivary glands (Sg), gut, leg, ovaries (Ov), testes (Te), and fat body (Fb). Data are presented as mean ± standard error of the mean (SEM), with *n* = 4. Different letters indicate statistically significant differences (one-way ANOVA, LSD, *p < 0.05*).

To investigate the expression levels of *NlCPSF30* across different developmental stages of *N. lugens*, we performed qRT-PCR using the total RNA extracted from whole bodies of first- to fifth-instar nymphs, as well as 1- to 3-day-old male and female adults. The results demonstrated that *NlCPSF30* is expressed throughout all developmental stages (Figure 3A). Notably, the highest expression level was observed in female adults, while the lowest was detected in second-instar nymphs.

For tissue-specific expression analysis, qRT-PCR was conducted on RNA extracted from various tissues, including the antennae, salivary glands, gut, legs, ovaries, and fat body of female adults, as well as the testes of male adults. The analysis revealed that *NlCPSF30* is expressed in multiple tissues of *N. lugens* (Figure 3B). The highest transcript level was detected in the fat body, while the salivary glands and legs exhibited the lowest expression among the tissues examined. These spatiotemporal expression patterns suggest that *NlCPSF30* plays a crucial role in the growth and development of *N. lugens*.

### 3.4. Effect of NlCPSF30 by RNAi on the Survival of BPH

**Figure 4:**
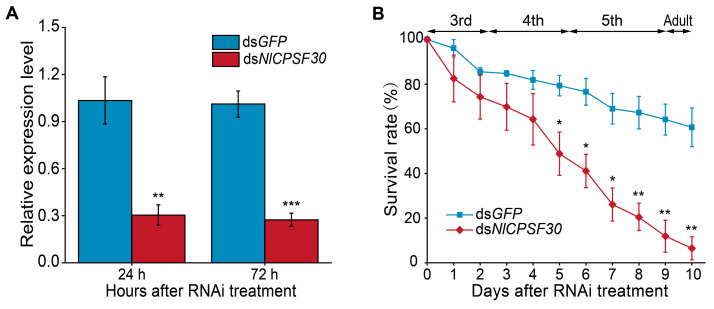
Effects of ds*NlCPSF30* interference on brown planthopper survival. (**A**) Assessment of RNAi efficiency following ds*NlCPSF30* injection. (**B**) Survival rates of third-instar brown planthopper nymphs post-microinjection of ds*NlCPSF30*. The progression from the third-instar to the adult stage in the ds*GFP* control group is illustrated in (**B**). Notably, compared to the ds*GFP* control group, BPH treated with ds*NlCPSF30* exhibited a slower developmental rate during the fifth-instar stage and did not reach adulthood before dying. Experiments were performed in triplicate, and data are presented as the mean ± standard error of the mean (SEM). Statistical significance is indicated as follows: * *p* < 0.05; ** *p* < 0.01; *** *p* < 0.001, determined using Student’s *t*-test.

To evaluate the effect of the *NlCPSF30* gene knockdown on the viability of the brown planthopper, we employed an RNAi approach via microinjection in third-instar nymphs. After 24 and 72 h of ds*NlCPSF30* injection, the expression levels of *NlCPSF30* were significantly reduced to 30.36% and 27.43%, respectively, compared to the ds*GFP* control group (Figure 4A). These results demonstrate that ds*NlCPSF30* effectively suppressed *NlCPSF30* expression relative to the control.

Following the silencing of *NlCPSF30*, we monitored the survival of brown planthoppers every 24 h (Figure 4B). The survival rate of the brown planthoppers treated with ds*NlCPSF30* dropped sharply from 82.54% on day 1 to 6.48% on day 10. In contrast, the survival rate of the nymphs injected with ds*GFP* declined more gradually, from 96.08% to 60.65%. From day 5 onward, the survival rate of the ds*NlCPSF30*-treated brown planthoppers was significantly lower than that of the ds*GFP* control group, with the disparity between the two groups progressively increasing. Notably, the majority of mortality in the brown planthoppers treated with ds*NlCPSF30* occurred during the late fourth-instar and fifth-instar developmental stages (Figure 4B). Additionally, none of the nymphs were able to successfully undergo the transition into adults.

**Figure 5:**
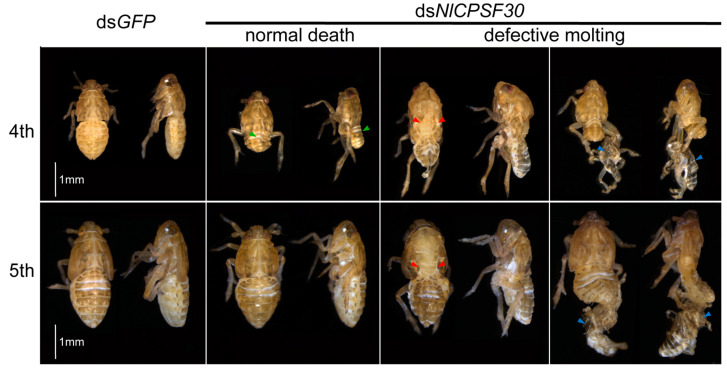
Observation of lethal phenotypes in *N. lugens* following dsRNA microinjection. Third-instar *N. lugens* nymphs were injected with ds*NlCPSF30* (treatment group) and ds*GFP* (control group), and the mortality phenotypes were recorded daily. In the treatment group, some nymphs exhibited molting failure, with varying degrees of molting difficulty observed from the fourth- to the fifth-instar stages. In the fourth-instar stage, the green arrows were used to denote normal death characterized by abnormal abdomen size. Additionally, two distinct defective molting phenotypes are illustrated by the red and blue arrows. The red arrows indicate the splitting of old cuticles at the notum, thereby exposing the underlying notum. Meanwhile, the blue arrows highlight instances where the old cuticle remains attached to the BPH’s abdomen and hind legs. Scale bars: 1 mm.

Following the microinjection of ds*NlCPSF30*, two distinct lethal phenotypes were observed: normal death and defective molting (Figure 5). Among the dead BPHs, more than half exhibited normal death, while the remainder displayed defective molting. In the case of normal death, the ds*NlCPSF30*-treated fourth-instar nymphs exhibited smaller body sizes, particularly a reduced abdomen size, compared to the ds*GFP* control group (Figure 5, indicated by green arrows). However, no significant difference in body size was observed between the ds*NlCPSF30*-treated fifth-instar nymphs and the ds*GFP* control group. Regarding the defective molting, two distinct phenotypes were identified. Firstly, there was a splitting of the old cuticles at the notum, resulting in the exposure of the underlying notum (Figure 5, indicated by red arrows). Secondly, the old cuticle remained attached to the BPH’s abdomen and hind legs, leading to unsuccessful molting (Figure 5, indicated by blue arrows). These observations collectively indicate that the knockdown of *NlCPSF30* severely disrupts the development of *N. lugens*.

### 3.5. The Impact of NlCPSF30 Knockdown on the Expression of Hormone-Related Genes

**Figure 6:**
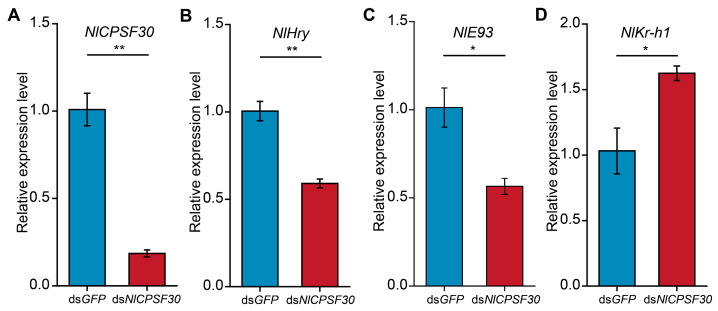
The change in expression hormone-related genes following *NlCPSF30* knockdown. The relative expression levels of *NlCPSF30* (**A**), *NlHry* (**B**), *NlE93* (**C**), and *NlKr-h1* (**D**) at 6 days post-injection of dsRNA. Third-instar nymphs were injected with ds*NlCPSF30* or ds*GFP* (control group). Experiments were performed in triplicate, and data are presented as the mean ± standard error of the mean (SEM). Statistical significance is indicated as follows: * *p* < 0.05; ** *p* < 0.01, determined using Student’s *t*-test.

In order to investigate the underlying molecular mechanisms behind the observed mortality and developmental defects in *N. lugens* resulting from silencing *NlCPSF30* expression, we focused on the hormonal pathways that regulate molting and metamorphosis. These processes are tightly controlled by ecdysone and juvenile hormone (JH), which are key hormones in insect development. To assess the impact of *NlCPSF30* knockdown on these pathways, we examined the expression levels of three important hormonal regulatory genes: *NlHry* and *NlE93*, both of which are ecdysone-responsive genes [33,34], and *NlKr-h1*, a juvenile hormone-responsive gene [35]. Six days after injecting third-instar nymphs with dsRNA, *NlCPSF30* expression was found to be reduced by 81% in the ds*NlCPSF30* treatment compared to the ds*GFP* control (Figure 6A), confirming the effectiveness of the knockdown. Similarly, the expression levels of the genes involved in the ecdysone signaling pathway, specifically *NlHry* (Figure 6B) and *NlE93* (Figure 6C), were reduced by 41% and 43%, respectively. These findings suggest that the molting process may be impaired. The downregulation of these two genes correlates with the observed molting defects in ds*NlCPSF30*-treated nymphs. Conversely, the expression of the JH pathway gene, *NlKr-h1*, was upregulated by 62% (Figure 6D), indicating that the development stage remains in the nymph state. This finding is consistent with the observation that none of the nymphs were able to successfully undergo the transition into adulthood. Overall, these results provide further insight into the molecular mechanisms affected by *NlCPSF30* knockdown, shedding light on the connection between *NlCPSF30* and the hormonal pathways governing molting and metamorphosis in *N. lugens*.

## 4. Discussion

In this study, we identified and characterized the *NlCPSF30* gene based on its homology with the mammalian CPSF30 and the brown planthopper genome. The NlCPSF30 protein is highly conserved across various organisms, containing five CCCH zinc-finger domains and two CCHC zinc-knuckle domains, critical for recognizing RNA substrates and processing pre-mRNA [21]. The RNAi-mediated knockdown of *NlCPSF30* resulted in increased mortality and impaired development in the nymphs of *N. lugens*. Additionally, it influenced the expression of genes related to hormone regulation, suggesting its involvement in RNA processing and gene regulation in this insect species.

CPSF30 is a zinc-finger protein with conserved zinc-finger and zinc-knuckle motifs, essential for its pre-mRNA 3′ end processing function. However, the number of zinc-finger and zinc-knuckle motifs varies among organisms. For instance, plants like *Arabidopsis thaliana* (AtCPSF30) possess three zinc-finger motifs [23], while five CCCH zinc-finger motifs are predominantly found in eukaryotic CPSF30 homologs, including the *Drosophila melanogaster* clipper (CLP) [17], yeast Yth1 [36], zebrafish Nar [37], and human CPSF30 [38]. Functional and structural studies indicate that the ZF2-ZF3 motifs of human CPSF30 are involved in recognizing the AAUAAA polyadenylation signal (PAS), while the ZF4-ZF5 motifs interact with hFip1, another CPSF subunit, with ZF4 showing a higher affinity for hFip1 [21,22,25]. In this study, ZF2-ZF4 of NlCPSF30 demonstrated high conservation across different insect orders (Figure 1), suggesting similar functions in RNA binding and pre-mRNA 3′ end processing.

The number of zinc-knuckle motifs also varies among CPSF30 homologs. Plant and yeast homologs lack zinc-knuckle motifs [23,36], while mammalian homologs possess one [25], and insect homologs, including CLP [17] and NlCPSF30 (this study), contain two. Zinc knuckles preferentially bind to the U-rich sequences within pre-mRNA, facilitating CPSF30’s association with PAS [21,25,39]. Thus, the zinc-finger and zinc-knuckle motifs of CPSF30 provide dual RNA recognition capabilities, crucial for its complex gene regulatory roles.

Our phylogenetic analysis demonstrated that NlCPSF30 is most closely related to the *Halyomorpha halys* CPSF30, aligning with their close taxonomic relationship within Hemiptera. This evolutionary proximity suggests functional similarities between CPSF30 proteins in these hemipteran pests, offering insights for developing RNAi-based pest control strategies.

The expression analysis at different developmental stages revealed that the highest levels of *NlCPSF30* were observed in adult females. This finding is consistent with previous studies in *D. melanogaster*, where the mRNA of the *CLP* gene was predominantly abundant in adult females [17,27]. Furthermore, a tissue-specific expression pattern demonstrated that *NlCPSF30* exhibited high expression in the fat body, a multifunctional tissue involved in molting regulation, hormone synthesis, energy metabolism, and reproduction. Silencing the expression of *NlCPSF30* led to defective molting, as shown in Figure 5. Moreover, our experimental observations revealed that nymphs treated with ds*NlCPSF30* displayed decreased movement activity in comparison to the ds*GFP* control group. In previous studies on the functional characterization of genes related to the BPH, it was found that *NlML1* (*myeloid differentiation factor 2 (MD-2)-related lipid-recognition 1*) [40] and *NlSPARC* (*Secreted protein*, *Acidic and Rich in Cysteine*) [41] were highly expressed in the fat body. Knockdown of these genes resulted in defective molting and increased mortality, which is consistent with the phenotypic effects observed upon silencing *NlCPSF30* expression. These results strongly suggest that *NlCPSF30* plays a crucial role in the development and regulation of essential physiological processes.

The evaluation of RNAi efficiency in gene function research is of utmost importance. It has been observed that the expression levels of the target gene exhibit variation over time after dsRNA injection and also vary with different doses of injected dsRNA. In our study, the expression levels of *NlCPSF30* were found to decrease by 70% and 73% at 24 h and 72 h post-injection with ds*NlCPSF30*, respectively (Figure 4A). A similar trend was observed in the investigation of the *lipophorin receptor* (*NlLpR*) gene, with expression levels decreasing by approximately 50% and 70% at 24 h and 72 h post-injection, respectively [42]. These findings suggest that the reduction in expression levels is more significant with a longer duration of post-injection dsRNA. Additionally, when the doses of ds*NlCPSF30* were adjusted to 11.5 ng, 23 ng, and 46 ng per insect, the expression levels of *NlCPSF30* decreased by 66%, 70%, and 77% at 24 h post-injection, respectively (Figure S2A). A similar pattern was observed in the examination of the autophagy-related gene *NlATG3* [29]. These results indicate that the effectiveness of RNAi is influenced by the dosage of dsRNA.

The knockdown of *NlCPSF30* resulted in a significant increase in mortality among *N. lugens*, particularly affecting normally developed insects (as shown in Figure 5). This effect was most prominent in fourth-instar nymphs, which exhibited smaller body sizes. Interestingly, this phenotype resembled the consequences of silencing certain genes from the fatty acid elongases (*ELOs*) family [43]. Additionally, the knockdown of *NlCPSF30* led to a defective molting phenotype, a characteristic observed in other RNAi-mediated injection experiments involving genes such as the chitinase-like gene family [44] and hormone-related [33,34,35] genes of BPH. Notably, the phenotypes resulting from the knockdown of five chitinase-like gene family genes (*NlCht1*, *NlCht5*, *NlCht7*, *NlCht9*, and *NlCht10*) [44] resembled those observed after injecting ds*NlCPSF30* (as depicted in Figure 5). To explore the underlying mechanisms, the expression of hormone-related genes like *NlHry* [33], *NlE93* [34], and *NlKr-h1* [35] were examined after injecting ds*NlCPSF30* in this study (as depicted in Figure 6). Previous studies have reported that knockdown of these genes resulted in defective molting, suggesting that changes in hormone-related genes, particularly *NlHry*, *NlE93*, and *NlKr-h1*, may be associated with the function of *NlCPSF30*.

The high mortality rate observed following the *NlCPSF30* knockdown underscores its potential as a target for RNAi-based pest management strategies. As discussed in the introduction, two strategies have been employed for utilizing RNAi-based pest control. Previous studies on plant-mediated RNAi in rice for controlling BPH have reported significant reductions in survival rates of BPH when using dsRNA transgenic rice plants that introduced the *ecdysone receptor* (*NlEcR*) [45] and *salivary protein 1* (*NlSP1*) [46] genes of BPH. Additionally, a novel delivery method for dsRNA of *chitin synthetase A* (*CHSA*) [47] has been successfully applied against BPH. These findings suggest that *NlCPSF30* holds great potential for practical use in controlling BPH in the future. However, before utilizing the *NlCPSF30* gene for BPH control, it is crucial to consider the dose-response effect, potential off-target and non-target effects, and potential resistance development.

Firstly, the dose-response effect plays a crucial role in RNAi efficacy [48]. In our study investigating the dosage of dsRNA administered to BPH, we observed a wide range of injection doses, spanning from a maximum of 3000 ng [49] to a minimum of 10 ng [50] per BPH. For our study, we opted for a relatively low dose, using a concentration of 1000 ng/µL and injecting 23 nL per BPH, resulting in a total dose of 23 ng. Additionally, we conducted supplementary experiments using injection doses of 11.5 ng (ds*NlCPSF30* 500 ng/µL) and 46 ng (ds*NlCPSF30* 2000 ng/µL) to assess the impact of different ds*NlCPSF30* doses on BPH survival (Figure S2). The results of these experiments showed a significant reduction in *NlCPSF30* gene expression across all three concentrations, and similar trends in mortality were observed, with higher concentrations leading to a more rapid decline in survival rates (Figure S2). These findings were consistent with the studies on BPH *NlATG3* [29] and the egg-specific gene *Nllet1* [50]. Overall, these findings make a valuable contribution to our understanding of the dose-response effects of dsRNA on BPH and offer valuable insights into the potential field applications of dsRNA for effective pest management.

Secondly, it is essential to address the potential off-target and non-target effects that can significantly impact the efficacy of RNAi in pest management applications. It is crucial to consider these effects and prevent any adverse consequences before implementing RNAi on a larger scale [51,52,53,54]. To achieve this, we conducted a multiple alignment by comparing the *NlCPSF30* nucleotide sequence with homologous sequences from other insect species. The alignment results revealed that the nucleotide sequence corresponding to the ZF domains exhibited a higher sequence identity compared to other regions. To minimize potential off-target and non-target effects of the *NlCPSF30* gene, it is recommended to select unique or BPH-specific regions of *NlCPSF30* as the target for dsRNA. However, it is important to note that further research is necessary to gain a more comprehensive understanding of these potential off-target and non-target effects.

Finally, resistance development has the potential to impact the efficiency of RNAi. While there have been no reported instances of resistance against RNAi in hemipteran pests, the establishment of the first dsRNA-resistant insect population through laboratory selection in western corn rootworm serves as evidence that resistance to dsRNA can potentially evolve after prolonged and extensive application of RNAi biopesticides [55]. This finding highlights the challenges associated with RNAi-based insect control for hemipteran pests and other pest orders. In the case of sap-feeding hemipteran pests, there are three identified potential mechanisms for resistance development: target modification, mutations in the core RNAi machinery, and physiological mutations [52]. These mechanisms further underscore the need for a comprehensive understanding of resistance development to develop effective strategies for RNAi-based pest control.

## 5. Conclusions

This study demonstrates that *NlCPSF30* is essential for the survival and development of brown planthoppers. RNAi-mediated knockdown of *NlCPSF30* resulted in a significant decrease in survival rates and the occurrence of molting defects, particularly during the late fourth-instar and fifth-instar developmental stages. Furthermore, it had an influence on the expression of the genes associated with hormone regulation. These results suggest that *NlCPSF30* is a promising target for RNAi-based pest control strategies. Future studies should focus on the development of transgenic rice expressing dsRNA targeting *NlCPSF30* or explore the feasibility of utilizing sprayable ds*NlCPSF30* biopesticides. Such innovations could provide a durable and environmentally friendly solution for managing brown planthopper infestations. Consequently, disrupting the expression of this key gene holds the potential to offer a sustainable alternative to conventional pest control measures.

## Figures and Tables

**Table 1:** Primers related to cloning and functional studies of *NlCPSF*30 gene.

Primer Name	Sequence (5′–3′)	Purpose	T_a_ (°C)	Length (bp)
*NlCPSF30*-F	TTAGTCAAGATTCCAGGTT	gene cloning	55	1614
*NlCPSF30*-R	CATCCCATTCATCAGTTT			
q*NlCPSF30*-F	TTACAAAGGGCACTACGC	qRT-PCR	60	97
q*NlCPSF30*-R	CTGTTGCTGCTGCTGGTT			
q*18S rRNA*-F	CGCTACTACCGATTGAA		60	131
q*18S rRNA*-R	GGAAACCTTGTTACGACTT			
ds*NlCPSF30-*F	TAATACGACTCACTATAGGGCGTGGCGACAGGACAATA	dsRNA synthesis	55	342
ds*NlCPSF30-*R	TAATACGACTCACTATAGGGAGTAGCAGGAAGTTCAAAA			
ds*GFP-*F	TAATACGACTCACTATAGGGAGAATGAGTAAAGGAGAAGAACTTTTC		55	495
ds*GFP-*R	TAATACGACTCACTATAGGGAGATTTGTATAGTTCATCCATGCCATGT			

T_a_: annealing temperatures; length: amplicon length.

## Data Availability

The data presented in this study are available in the article.

## Supplementary Materials

The following supporting information can be downloaded at: https://www.mdpi.com/article/10.3390/insects15110860/s1, Table S1: List of orthologs of CPSF30 protein in multiple sequence alignment and phylogenetic analysis. Table S2: List of orthologs showing the amino acid identity of NlCPSF30 protein in multiple sequence alignment. Figure S1: A phylogenetic tree was constructed using NlCPSF30 and 30 orthologs from six different insect orders. The species from each insect order were represented by curved frames of different colors. Specifically, the curved frames colored in red, orange, pink, green, blue, and purple represented species from the Hymenoptera, Orthoptera, Hemiptera, Lepidoptera, Coleoptera, and Diptera orders, respectively. Figure S2: Effects of different concentrations of ds*NlCPSF30* on the survival rate of *N. lugens*. (**A**) Relative expression levels of *NlCPSF30* were measured 24 h after the injection of ds*NlCPSF30* at three different concentrations: ds*NlCPSF30* (500 ng/μL), ds*NlCPSF30* (1000 ng/μL), and ds*NlCPSF30* (2000 ng/μL) into third-instar nymphs. As a control, ds*GFP* (1000 ng/μL) treatment was used. The relative expression levels of the gene were normalized using *Nl18S* as the reference gene. (**B**) Survival rates of the nymphs were assessed following the injection with ds*GFP* (1000 ng/μL), ds*NlCPSF30* (500 ng/μL), ds*NlCPSF30* (1000 ng/μL), and ds*NlCPSF30* (2000 ng/μL). Each treatment was replicated three times, with 30–40 brown planthoppers in each replicate. The data are presented as mean ± SEM from three independent experiments. Statistical analysis was performed using a two-sided Student’s *t*-test to determine significant differences (* *p* < 0.05; ** *p* < 0.01; *** *p* < 0.001).
