# Supplementary material for: Functional Analysis of CPSF30 in Nilaparvata lugens Using RNA Interference Reveals Its Essential Role in Development and Survival
Source: Insects. 2024 Nov 3;15(11):860. doi: 10.3390/insects15110860 (PMC11594811; doi:10.3390/insects15110860)
Supplement: Supplementary file 1 [file insects-15-00860-s001.zip › Table S1 CPSF30 proteins.pdf]

Table S1: List of orthologs of CPSF30 protein in multiple sequence alignment and phylogenetic analysis

Number	Species	GenBank accession number	Class/order
1	*Nilaparvata lugens*	XP_022200280	Insecta/Hemiptera
2	*Halyomorpha halys*	XP_014284934	Insecta/Hemiptera
3	*Bemisia tabaci*	XP_018898339	Insecta/Hemiptera
4	*Daktulosphaira vitifoliae*	XP_050533717	Insecta/Hemiptera
5	*Myzus persicae*	XP_022172674	Insecta/Hemiptera
6	*Aphis gossypii*	XP_027853365	Insecta/Hemiptera
7	*Diorhabda carinulata*	XP_057665302	Insecta/Coleoptera
8	*Anoplophora glabripennis*	XP_018576327	Insecta/Coleoptera
9	*Leptinotarsa decemlineata*	XP_023022873	Insecta/Coleoptera
10	*Cylas formicarius*	XP_060519535	Insecta/Coleoptera
11	*Dendroctonus ponderosae*	XP_019754034	Insecta/Coleoptera
12	*Sitophilus oryzae*	XP_030761179	Insecta/Coleoptera
13	*Bombyx mori*	NP_001040511	Insecta/Lepidoptera
14	*Bombyx mandarina*	XP_028040942	Insecta/Lepidoptera
15	*Galleria mellonella*	XP_026762580	Insecta/Lepidoptera
16	*Spodoptera frugiperda*	XP_035445348	Insecta/Lepidoptera
17	*Helicoverpa armigera*	XP_021189422	Insecta/Lepidoptera
18	*Helicoverpa zea*	XP_047026520	Insecta/Lepidoptera
19	*Drosophila melanogaster*	NP_477156	Insecta/Diptera
20	*Zeugodacus cucurbitae*	XP_011184594	Insecta/Diptera
21	*Drosophila ananassae*	XP_014762287	Insecta/Diptera
22	*Anabrus simplex*	XP_067000942	Insecta/Orthoptera
23	*Schistocerca americana*	XP_046994283	Insecta/Orthoptera
24	*Schistocerca gregaria*	XP_049861155	Insecta/Orthoptera
25	*Schistocerca piceifrons*	XP_047112714	Insecta/Orthoptera
26	*Schistocerca nitens*	XP_049808070	Insecta/Orthoptera
27	*Apis florea*	XP_003698318	Insecta/Hymenoptera
28	*Nasonia vitripennis*	XP_001604679	Insecta/Hymenoptera
29	*Vespa crabro*	XP_046826437	Insecta/Hymenoptera
30	*Solenopsis invicta*	XP_011166635	Insecta/Hymenoptera
31	*Apis cerana*	XP_028519923	Insecta/Hymenoptera
32	*Symphalangus syndactylus*	XP_063502265	Mammal
33	*Homo sapiens*	NP_001305090	Mammal
34	*Tupaia chinensis*	ELW64885	Mammal
35	*Aotus nancymaae*	XP_012310940	Mammal
36	*Rattus norvegicus*	NP_001388044	Mammal

37	*Mus musculus*	NP_001361645	Mammal
38	*Arabidopsis thaliana*	NP_001319113	Plant
39	*Artemisia annua*	PWA56346	Plant
40	*Sesamum radiatum*	KAL0305431	Plant
41	*Sesamum alatum*	KAK4421049	Plant
42	*Perilla frutescens var. hirtella*	KAH6826237	Plant
43	*Sesamum latifolium*	KAL0442454	Plant
