# Supplementary material for: Functional Analysis of CPSF30 in Nilaparvata lugens Using RNA Interference Reveals Its Essential Role in Development and Survival
Source: Insects. 2024 Nov 3;15(11):860. doi: 10.3390/insects15110860 (PMC11594811; doi:10.3390/insects15110860)
Supplement: Supplementary file 1 [file insects-15-00860-s001.zip › Table S2 the amino acid identity of NlCPSF30 protein.pdf]

Table S2: List of orthologs showing the amino acid identity of NlCPSF30 protein in multiple sequence alignment

Number	Species	GenBank accession number	Amino acid identity
1	*Halyomorpha halys*	XP_014284934	62.07%
2	*Anabrus simplex*	XP_067000942	61.49%
4	*Bombyx mori*	NP_001040511	56.10%
4	*Diorhabda carinulata*	XP_057665302	54.65%
5	*Apis florea*	XP_003698318	57.56%
6	*Drosophila melanogaster*	NP_477156	51.45%
