# Supplementary figures and images for: Functional Analysis of CPSF30 in Nilaparvata lugens Using RNA Interference Reveals Its Essential Role in Development and Survival
Source: Insects. 2024 Nov 3;15(11):860. doi: 10.3390/insects15110860 (PMC11594811; doi:10.3390/insects15110860)

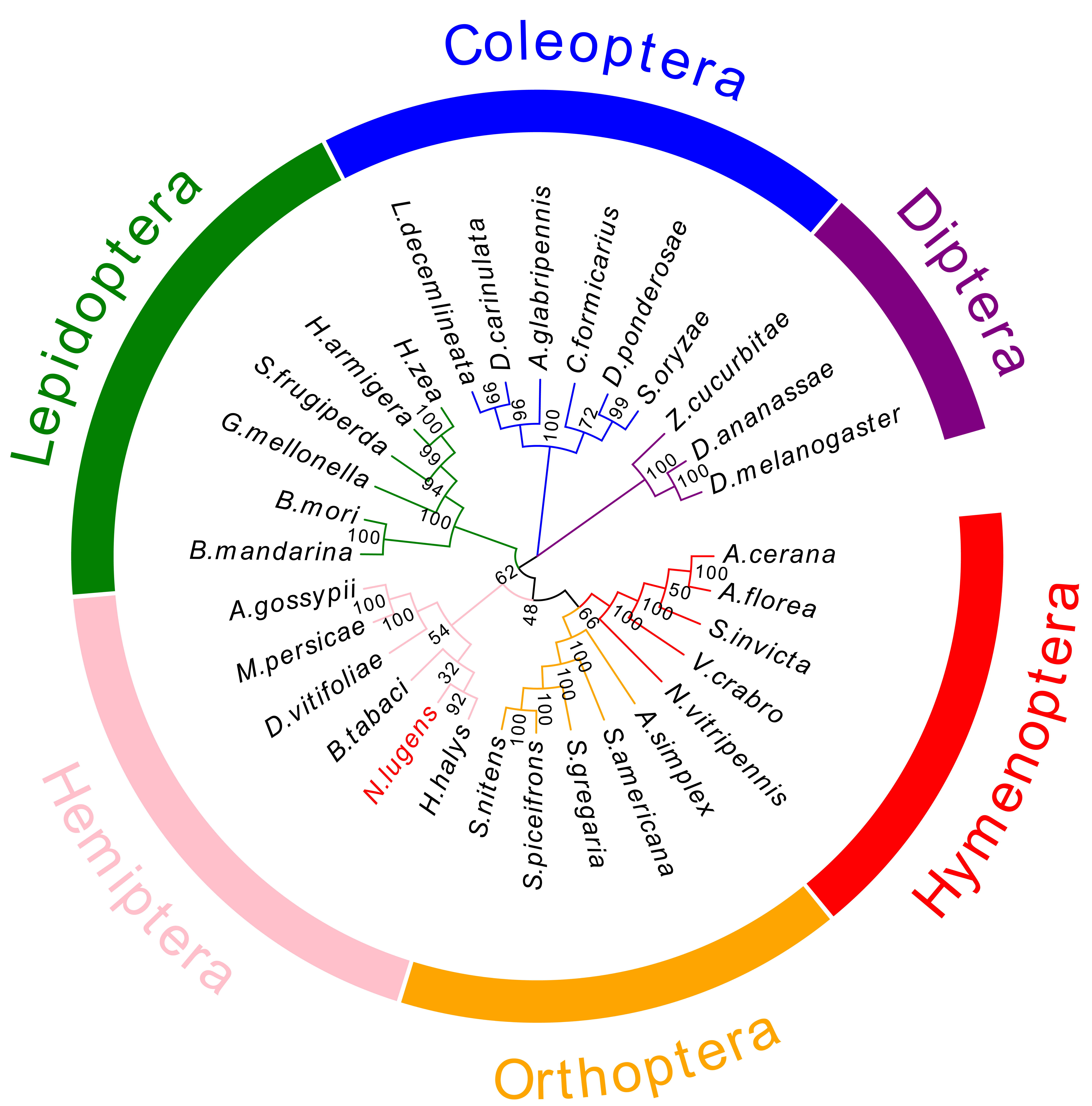

Supplement: Supplementary file 1 [file insects-15-00860-s001.zip › Figure S1.jpg]

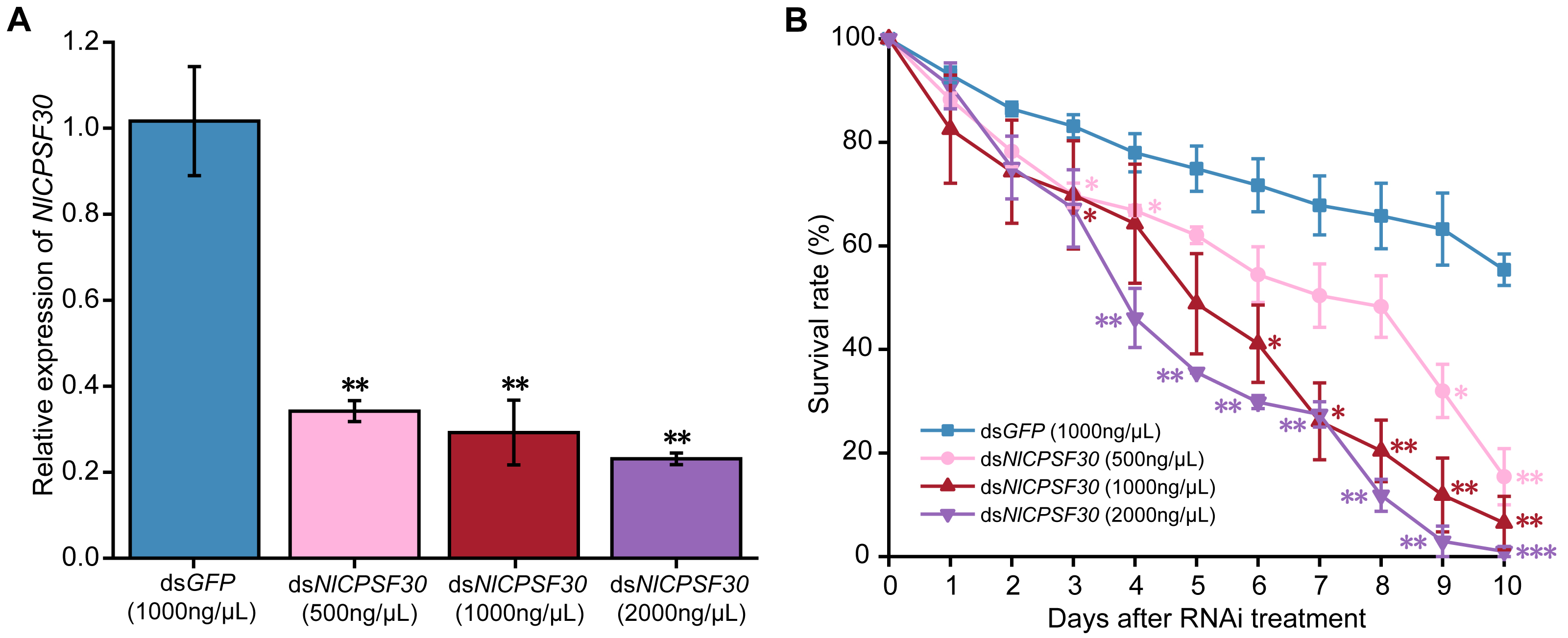

Supplement: Supplementary file 1 [file insects-15-00860-s001.zip › Figure S2.jpg]
